# Targeted RNAi screen identifies transcriptional mechanisms that prevent premature degeneration of adult photoreceptors

**DOI:** 10.3389/freae.2023.1187980

**Published:** 2023-05-05

**Authors:** Spencer E. Escobedo, Sarah E. McGovern, Juan P. Jauregui-Lozano, Sarah C. Stanhope, Paul Anik, Kratika Singhal, Ryan DeBernardis, Vikki M. Weake

**Affiliations:** ^1^ Department of Biochemistry, Purdue University, West Lafayette, IN, United States; ^2^ Purdue University Center for Cancer Research, Purdue University, West Lafayette, IN, United States

**Keywords:** *Drosophila*, eye, aging, photoreceptors, RNAi screen, retinal degeneration, epigenetic

## Abstract

Aging is associated with a decline in visual function and increased prevalence of ocular disease, correlating with changes in the transcriptome and epigenome of cells in the eye. Here, we sought to identify the transcriptional mechanisms that are necessary to maintain photoreceptor viability and function during aging. To do this, we performed a targeted photoreceptor-specific RNAi screen in *Drosophila* to identify transcriptional regulators whose knockdown results in premature, age-dependent retinal degeneration. From an initial set of 155 RNAi lines each targeting a unique gene and spanning a diverse set of transcription factors, chromatin remodelers, and histone modifiers, we identified 18 high-confidence target genes whose decreased expression in adult photoreceptors leads to premature and progressive retinal degeneration. These 18 target genes were enriched for factors involved in the regulation of transcription initiation, pausing, and elongation, suggesting that these processes are essential for maintaining the health of aging photoreceptors. To identify the genes regulated by these factors, we profiled the photoreceptor transcriptome in a subset of lines. Strikingly, two of the 18 target genes, *Spt5* and *domino*, show similar changes in gene expression to those observed in photoreceptors with advanced age. Together, our data suggest that dysregulation of factors involved in transcription initiation and elongation plays a key role in shaping the transcriptome of aging photoreceptors. Further, our findings indicate that the age-dependent changes in gene expression not only correlate but might also contribute to an increased risk of retinal degeneration.

## Introduction

The risk of ocular disease strongly increases with advanced age, particularly after age 75, leading to an increased prevalence of blindness and visual impairment irrespective of race or regional groups (Klaver et al., 1998; Klein and Klein, 2013). Moreover, aging is associated with an increased incidence of eye diseases such as cataracts, diabetic retinopathy, glaucoma, and age-related macular degeneration (AMD) (Coleman et al., 2008). Although environmental factors such as smoking, diet, and sunlight exposure can alter the risk of developing age-associated eye disease (Ng Yin Ling et al., 2021), chronological age remains the major factor that influences the likelihood of developing eye disease. We and others have identified reproducible and robust changes in gene expression that occur in aging cells within the eye (Parapuram et al., 2010; Hall et al., 2017). In mice, aging rod photoreceptors undergo global changes in gene expression that precede any signs of retinal degeneration (Swaroop et al., 2010; Campello et al., 2021). Age-regulated genes in rods are involved in morphogenesis, motor axon guidance, neuronal signaling, and regulation of transcription (Parapuram et al., 2010). *Drosophila* photoreceptors, which functionally resemble vertebrate rods, show similar changes in the aging transcriptome, with increased expression of DNA damage response genes and downregulation of genes involved in neuronal function that correlate with decreased visual function (Hall et al., 2017; Jauregui-Lozano et al., 2021).

In addition to changes in gene expression, there are age-dependent alterations to chromatin accessibility, histone marks, and DNA methylation in the eye. For example, changes in chromatin accessibility have been detected at the onset of the disease state for AMD (Wang et al., 2018) and in aging *Drosophila* photoreceptors (Jauregui-Lozano et al., 2023). Further, changes in DNA methylation are observed in aging mouse rods, particularly near genes involved in energy metabolism (Corso-Díaz et al., 2020). Similar broad changes in gene expression and chromatin marks are also observed in other aging tissues, suggesting that these transcriptional and epigenetic changes are a common feature of aging (Horvath, 2013; Pal and Tyler, 2016; Stegeman et al., 2018; López-Otín et al., 2023). The reproducible changes in some epigenetic marks such as DNA methylation in aging tissues have led to its use as an epigenetic clock to estimate the chronological age of specific cell types or in various disease states and cancer cell types (Horvath and Raj, 2018). Moreover, re-expressing selected Yamanaka transcription factors in mouse retinal ganglion cells restored a youthful pattern of DNA methylation, regenerative capacity, and visual function in older mice (Lu et al., 2020). Collectively, these data suggest that age-associated changes in gene expression patterns in the eye directly contribute to the increased risk of ocular disease with advanced age.

We reasoned that the reproducible epigenetic and transcriptional changes in aging photoreceptors suggested that the activity of specific transcriptional regulatory mechanisms declines with advanced age. If the age-dependent changes in photoreceptor gene expression contribute to an increased risk of retinal degeneration, then decreasing the expression of specific transcriptional regulators in adult photoreceptors should result in premature cell death. To test if disrupting specific transcriptional processes could lead to premature retinal degeneration, we performed a targeted RNAi screen in *Drosophila* photoreceptors. We show that knockdown of several epigenetic regulators associated with transcription elongation leads to premature age-dependent retinal degeneration and results in gene expression signatures that resemble much older flies. Our data suggest that diminished ability to induce activation of transcription may underly a large proportion of the changes in gene expression observed in aging photoreceptors. Moreover, our findings suggest that the age-dependent changes in gene expression in photoreceptors directly contribute to the increased risk of retinal degeneration.

## Results

### Characterization of age-dependent retinal degeneration in the RNAi screen background

We sought to perform a targeted RNAi-based candidate screen to identify the transcriptional and epigenetic mechanisms that are required for photoreceptor survival during aging. To do this, we generated flies in which we could express *UAS-shRNA* against various target genes in differentiated, adult photoreceptors using *Rh1-Gal4* in the presence of *UAS-Dcr* to enhance knockdown. These flies also express photoreceptor-specific luciferase (*Rh1-ffluc*), enabling us to assess photoreceptor survival throughout aging using two independent assays: luciferase activity as a proxy for photoreceptor number (Stegeman et al., 2018), and optic neutralization in live flies to assess rhabdomere integrity (Franceschini and Kirschfeld, 1971) (Figure 1A). To quantify rhabdomere loss by optic neutralization, we scored retinal degeneration from little to no degeneration as a score of 1 to highly degenerated ommatidia with a score of 7 (Figure 1B). We refer to these *Rh1-ffluc, Rh1-Gal4>UAS-Dcr2* flies hereafter as *Rh1-Gal4* for simplicity. Previous characterization by our lab has shown that the photoreceptor-specific *Rh1-Gal4* driver becomes effective in adult flies by 2 days post-eclosion so it does not impact eye development and remains effective throughout aging (Jauregui-Lozano et al., 2023).

**FIGURE 1:**
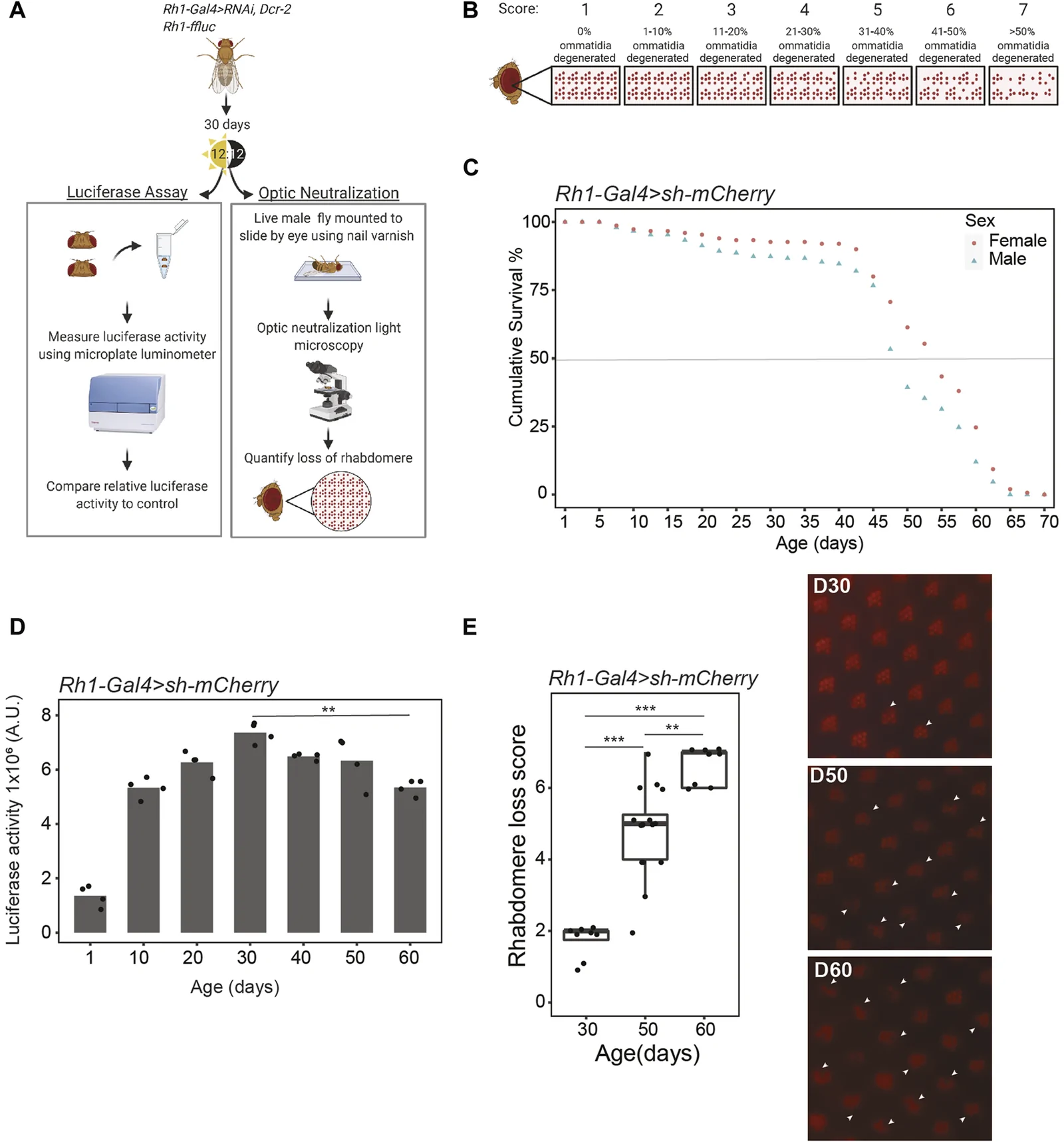
*Drosophila* undergo age-dependent retinal degeneration. **(A)** Schematic describing the techniques used to assess retinal degeneration in aging *Rh1-ffluc, Rh1-Gal4>shRNA Drosophila*. **(B)** Scoring scheme used to analyze optic neutralization images for the severity of rhabdomere loss using a scale of 1–7, where 7 indicates severe retinal degeneration. **(C)** Survival curve of *Rh1-ffluc, Rh1-Gal4>sh-mCherry* male and female flies (*n* = 300). **(D)** Luciferase activity (arbitrary units) in heads of aging flies, *p*-value < 0.005 (**), ANOVA with post-hoc Tukey HSD. **(E)** Optic neutralization at D30, D50, and D60. Scores are shown in the left panel (n ≥ 8 independent flies) with representative images shown in the right panel. Arrowheads indicate missing rhabdomeres. *p*-value (***< 0.005, ****<0.0005), ANOVA with posthoc Tukey HSD.

We then characterized photoreceptor survival throughout aging in the *Rh1-Gal4* screen background flies expressing RNAi against *mCherry*. This *sh-mCherry* line was subsequently used as one of the control lines for the RNAi screen. These flies show a similar lifespan to other wild-type *Drosophila* strains at 25°C with a median survival of 52 and 57 days for male and female flies, respectively (Figure 1C). Consistent with the continued increase in expression of Rh1 (Rhodopsin 1, encoded by the *ninaE* gene) in the first few days following eclosion, we observe a substantial increase in luciferase activity from day one (D1) to D10, followed by maintained levels of luciferase activity that only start to decline around D60 (Figure 1D). This decrease in luciferase activity at D60 correlates with substantial and significant rhabdomere loss as determined by optic neutralization at this same age (Figure 1E). In contrast, little to no retinal degeneration is observed by either technique at D30, with intermediate and highly variable rhabdomere loss at D50 (Figure 1E). We conclude that old *Rh1-Gal4* flies, defined as being in the second half of their median lifespan, exhibit significant retinal degeneration that is almost entirely absent from young (D10) or middle-aged flies (D30). We and others have previously shown that red-eyed *Drosophila* show negligible retinal degeneration before D40 (Hall et al., 2017).

### Photoreceptor-specific targeted RNAi screen reveals transcription elongation factors are necessary for age-dependent photoreceptor survival

Next, we crossed lines expressing RNAi against a variety of transcriptional regulators with the *Rh1-Gal4* flies, and assessed photoreceptor health by luciferase assays and optic neutralization at D30, when control flies exhibit little to no retinal degeneration (Figure 2A). We initially used RNAi lines that targeted 155 unique genes representing a diverse group of gene regulatory factors. 94 of these RNAi lines included histone modifiers, chromatin remodelers, and factors that regulate specific aspects of the transcription cycle (Figure 2B; Supplementary Table S1). We also targeted 61 transcription factors that were previously identified as having enriched binding motifs in the promoters of genes that were differentially expressed in aging photoreceptors (Hall et al., 2017). In addition, we used five independent RNAi lines that target genes not expressed in *Drosophila* as negative controls: mCherry, GFP #1, GFP #2, LexA #1, LexA #2.

**FIGURE 2:**
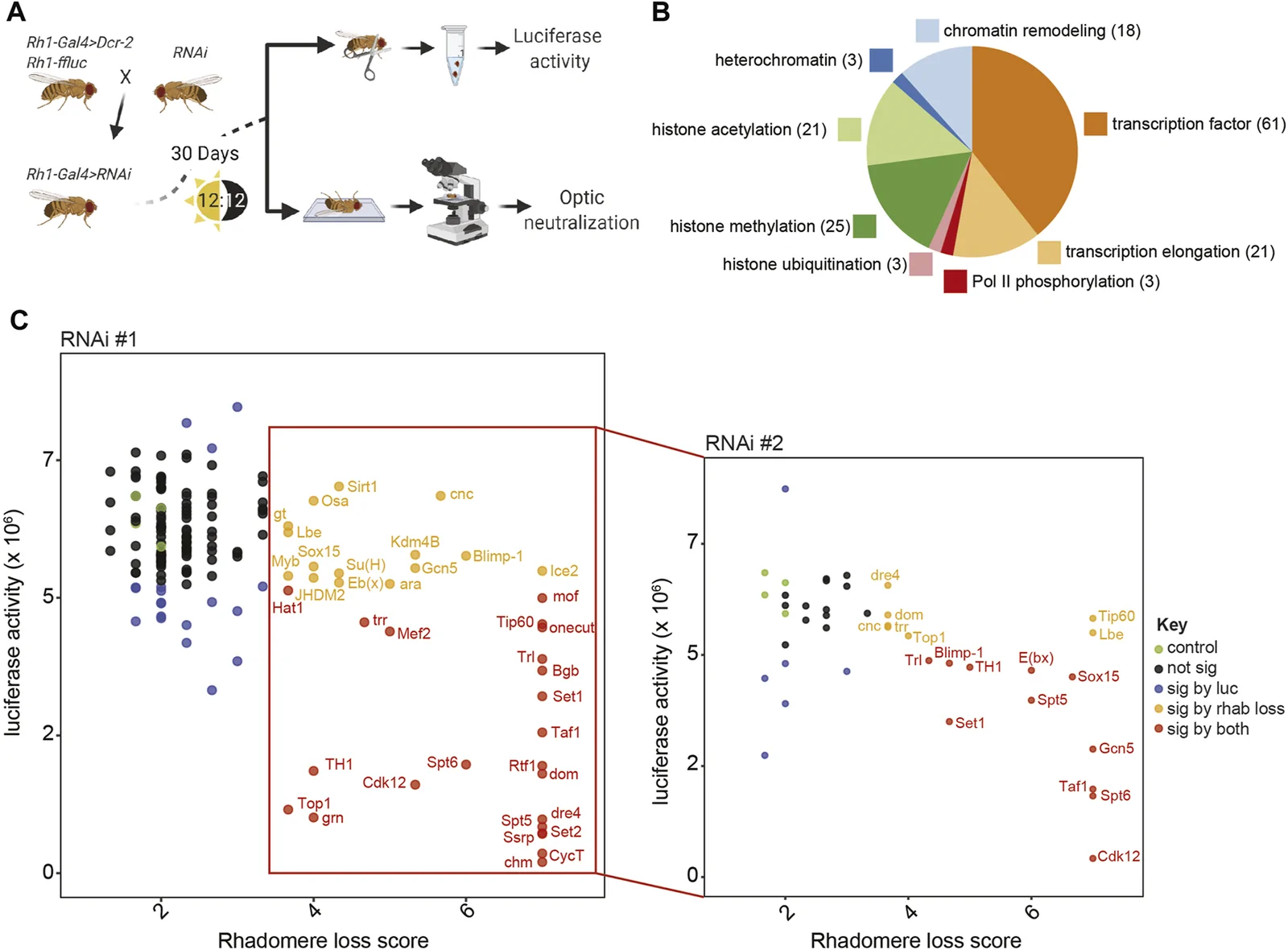
Targeted RNAi screen identifies 18 transcriptional regulators that are necessary for the survival of aging photoreceptors. **(A)** Schematic describing the targeted RNAi screen to identify factors that are necessary in adult photoreceptors for cell survival. **(B)** Pie chart showing the gene functions of the 155 unique genes tested in the targeted RNAi screen. **(C)** Scatter plot showing the mean luciferase activity versus optic neutralization score for each of the initial (RNAi #1, left panel) or secondary (RNAi #2, right panel) RNAi lines targeted (*n* = 3). Each point represents a single *shRNA* line and is colored as described in the legend (green, control; black, non-significant; blue, significant change in luciferase activity; orange, significant change in optic score; red, significant change in luciferase activity and optic score).

We primarily selected VALIUM20 lines from the Transgenic RNAi Project (TRiP) collection, but used TRiP VALIUM1/10 or Vienna *Drosophila* Stock Center (VDRC) KK RNAi collections if VALIUM20 lines were not available for the target gene (Dietzl et al., 2007). Whereas the VALIUM20 lines are *shRNA* transgenes that utilize the *mir-1* scaffold for efficient and specific knockdown of the target gene (Ni et al., 2011; Perkins et al., 2015), the VALIUM1/10 and KK RNAi lines utilize long dsRNA hairpins (Ni et al., 2008), which have an increased likelihood of off-target effects (Kulkarni et al., 2006). As expected from the control flies expressing RNAi against *mCherry* (Figures 1D, E), we did not observe retinal degeneration in four of the five control lines at D30 using luciferase assays or optic neutralization (Figure 2C, green circles). However, one of the lines expressing an RNAi against *GFP* (GFP #2) had substantial rhabdomere loss by optic neutralization and a significant decrease in luciferase activity, presumably due to off-target effects; we excluded this line as a control for this study (Supplementary Table S1).

We separately compared luciferase activity and optic neutralization scores between each of the 155 target gene RNAi lines and the remaining four controls and identified RNAi lines that showed significant changes in both luciferase activity and optic neutralization scores (*p* < 0.05, Dunnett’s test; 22 genes; red circles, Figure 2C left panel labeled genes). We also identified RNAi lines with significant changes only in luciferase activity (18 genes; blue circles) or optic neutralization scores (14 genes; yellow circles). We reasoned that changes in luciferase activity that were not accompanied by rhabdomere loss most likely represent an altered expression of the *Rh1-ffluc* transgene; thus, we focused on the 36 RNAi #1 targets with significant changes in optic scores for validation. To decrease the likelihood of false positives due to off-target effects, we tested an additional independent RNAi line for each of the 36 genes targeted by these lines (RNAi #2, right panel Figure 2C). Only 18 of these 36 independent RNAi lines resulted in significant degeneration phenotypes (red and yellow circles, RNAi #2, right panel Figure 2C); these 18 genes represent high confidence targets for factors that promote survival of adult photoreceptors during aging.

To test if the retinal degeneration observed at D30 for each of these 18 factors was due to the expression of the respective RNAi, we performed optic neutralization in each of these RNAi lines outcrossed to *Rh1-ffluc* in the absence of the *Rh1-Gal4* driver (Figure 3A, no driver). We did not observe substantial retinal degeneration in either the first or second RNAi line targeting the 18 high-confidence targets in these no-driver controls, indicating that photoreceptor-specific knockdown of the respective target gene indeed underlies the observed decrease in photoreceptor survival. To determine whether the retinal degeneration phenotype induced by photoreceptor-specific RNAi against the 18 high-confidence target genes was progressive with age, we next compared optic neutralization scores in both young (D10) and middle-aged (D30) flies. We found that the majority of the RNAi lines exhibit a retinal degeneration phenotype that becomes progressively worse with age (Figure 3B). We attribute differences in the progression of the retinal degeneration phenotype between the two different RNAi lines targeting each gene (e.g., *Spt5, Cdk12)* to potential differences in knockdown efficiency. Together, our data suggest that the proper expression of these 18 factors becomes increasingly important for regulating gene expression pathways that promote survival in old photoreceptors. In summary, our screen has identified 18 genes whose normal expression is necessary for photoreceptor survival in aging flies (Table 1).

**FIGURE 3:**
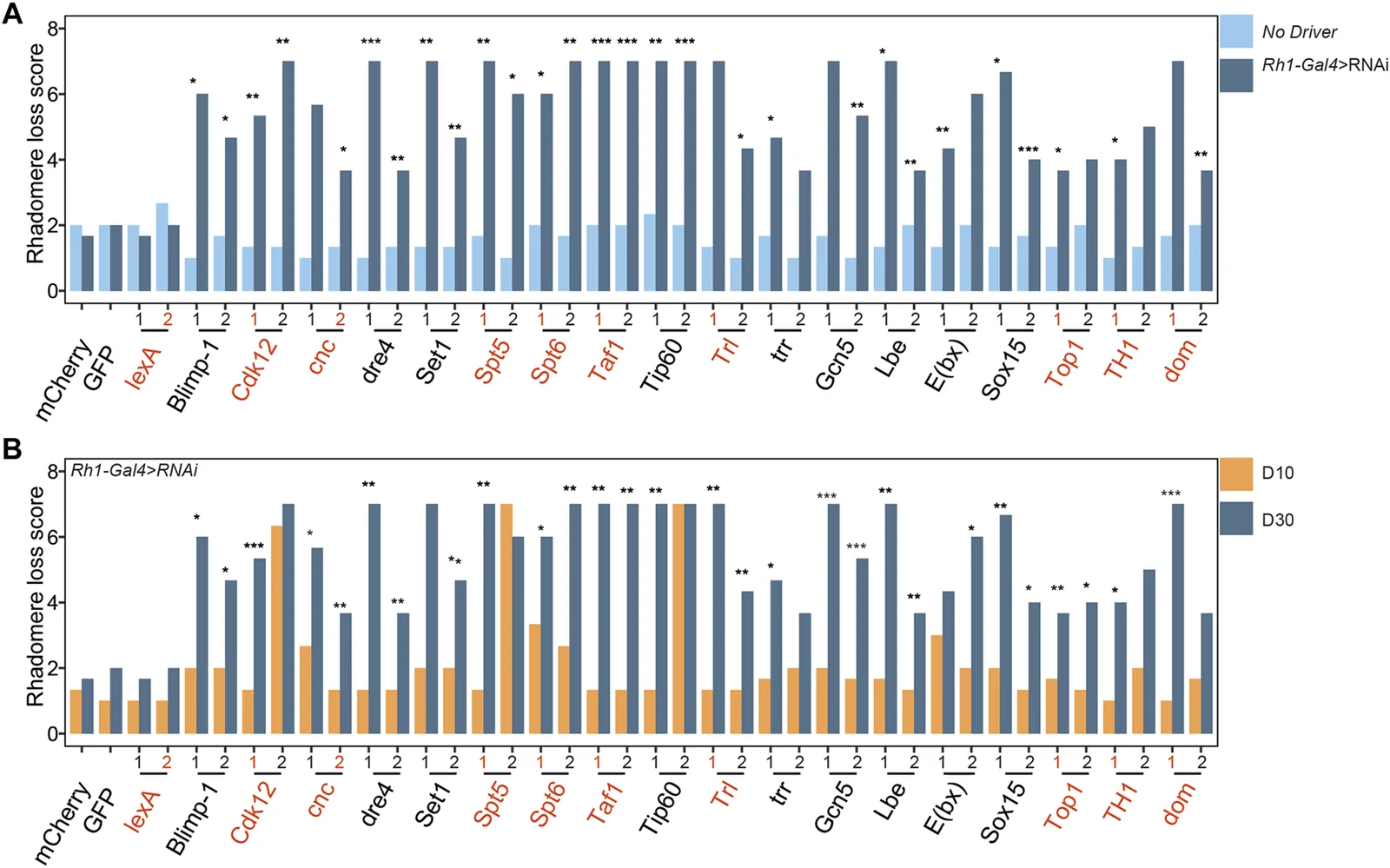
Retinal degeneration induced by knockdown of the 18 factors is progressive and requires Gal4 expression. **(A)** Bar plot showing mean optic neutralization scores in D30 flies for each *UAS-shRNA* line in the presence or absence of *Rh1-Gal4* (*n* = 3). #1 and #2 correspond to initial and secondary RNAi lines tested in the original screen. *p*-value (*<0.05, **<0.005, ***<0.0005), Students one-tailed *t*-test. **(B)** Bar plot showing mean optic neutralization scores in D10 versus D30 flies expressing *Rh1-Gal4>shRNA* against indicated targets. *p*-values as in panel A. RNAi lines used for subsequent RNA-seq analysis are labeled in red.

**TABLE 1:** 18 gene regulatory factors identified in RNAi screen that prevent premature retinal degeneration in adult photoreceptors.

Gene expression role		Gene	Gene function	Complex
Transcription Elongation	GAGA Factor	*Trl*	GAGA factor. Regulates gene expression through its role as both a transcription factor and chromatin regulator	
	Chromatin remodeler	*E(bx)*	Regulator of homeotic and heat shock gene expression, ISWI chromatin remodeling complex member	NURF
	Pausing Factors	*Spt5*	One of the two proteins that make up the dimeric DSIF complex. Regulates promoter proximal pausing and inducible gene expression	DSIF
		*TH1*	One of the four subunits of the NELF complex. Involved in inducible gene expression and Pol II promoter proximal pausing	NELF
	CTD kinase	*Cdk12*	Phosphorylates Serine 2 of Pol II CTD for Pol II elongation. Shown to regulate Nrf2 (Cnc) target genes in *Drosophila*	
	Histone Chaperone	*Spt6*	Elongation factor involved in nucleosome reassembly behind elongating Pol II	
		*dre4*	Histone chaperone like complex member, maintains promoter proximal paused Pol II	FACT
Transcription activation	Histone Acetyltransferase	*Tip60*	Histone acetyltransferase involved in H4 acetylation. for DNA damage and stress response	NuA4
		*Gcn5*	Histone acetyltransferase of the SAGA and ATAC complexes. cofactor for RNA Pol II transcription	SAGA/ATAC
	Histone Methyltransferase	*Set1*	Histone methyltransferase responsible for H3K4me3. Activates gene expression through modifications made at gene promoters	Compass
		*trr*	Histone methyltransferase responsible for H3K4me1.Regulates enhancer elements	Compass like/MLR
	RNA Pol II Cofactor	*Taf1*	Largest complex member of the TFIID complex. Targets TFIID to promoter. Mutations in humans associated with intellectual disabilities	TFIID
	Chromatin remodeler	*dom*	ATP-dependent chromatin remodeler involved in incorporating H2A.V. Recruited to sites of DNA damage Also involved in H4 acetylation. Involved in Notch signaling	SWR1 and NuA4
Topoisomerase		*Top1*	DNA topoisomerase. Relieves topological stress from DNA during replication and transcription	
Transcription Factors	Stress response	*cnc*	Nrf2 homolog. Activates oxidative stress/inducible gene expression	
	Development	*Blimp-1*	Prdm1 homolog. C2H2 TF. Involved in cellular response to ecdysone, as well as neuronal development in human cells	
		*lbe*	NK-like Homeobox TF. Roles in muscle and heart development	
		*Sox15*	HMGB TF. Maintains cell stemness	

We observed a striking enrichment for factors (7 of 18 factors) that are involved in transcription elongation and release of the paused RNA polymerase II (Pol II) among the RNAi screen high-confidence hits. First, we identified both the *Drosophila* GAGA factor (GAF) *Trl* and the Nucleosome Remodeling Factor (NURF) subunit *E(bx)*, which together are important for nucleosome depletion in gene promoters (Fuda et al., 2015; Tsai et al., 2016), recruiting Pol II (Judd et al., 2021), and regulating promoter-proximal pause release further downstream (Fuda et al., 2015). We also identified the DRB Sensitivity Inducing Factor (DSIF) complex member *Spt5* and the Negative elongation Factor (NELF) subunit *TH1*, both of which are important regulators of promoter-proximal pausing (Andrulis et al., 2000; Wu et al., 2003; Aoi et al., 2020). In addition, we identified the Pol II CTD Ser2 kinase *Cdk12,* and the histone chaperones *Spt6* and Facilitates Chromatin Transactions (FACT) complex subunit *dre4*, which all promote productive transcription elongation by Pol II (Orphanides et al., 1998; Andrulis et al., 2000; Tellier et al., 2020).

The next largest group of factors identified by our screen can be broadly characterized as regulators of transcription activation. These included the histone acetyltransferases (HAT) *Tip60* and *Gcn5* of the NuA4/Tip60 and SAGA/ATAC complexes, respectively. Interestingly, our screen also identified the chromatin remodeler *dom*, which encodes two splice isoforms that are either incorporated into the NuA4/Tip60 or SWR1-like complex (Squatrito et al., 2006; Scacchetti et al., 2020)*.* Notably, the *dom* RNAi #1 line used specifically targets the isoform that is associated with the SWR-1 like complex, suggesting that this complex might be the relevant target. We also identified two of the three H3K4 methyltransferases in flies (*Set1* and *trr*) as well as the TFIID subunit *Taf1* and the topoisomerase *Top1*.

Although transcription factors were overrepresented in the target screen (61/155 factors), these were underrepresented in the targets identified by our screen (Table 1). Only four genes encoding transcription factors were identified as being necessary for age-dependent photoreceptor survival: *cnc*, *Blimp-1*, *Lbe*, and *Sox15*. *Blimp-1* encodes the ortholog of mammalian *Prdm1*, which plays an important role in determining photoreceptor identity in the mouse retina (Brzezinski et al., 2010; Brzezinski et al., 2013). The transcription factor encoded by *cnc* is the ortholog of mammalian Nrf2, the master regulator of anti-oxidative and detoxification response (Vomund et al., 2017)*.* The homeobox transcription factor *Lbe* was originally described for its role in myogenesis (Jagla et al., 1997; Souidi and Jagla, 2021), but is also involved in neuronal differentiation and used as a marker for neuroblasts (Gabilondo et al., 2016; Urbach et al., 2016; Stratmann et al., 2019). *Sox15* encodes a transcription factor involved in both wing disc and mechanoreceptor development (Dichtel-Danjoy et al., 2009; Miller et al., 2009).

### Identification of gene expression changes induced by RNAi screen hits in photoreceptors

We previously showed that there is a correlation between increasing gene length and decreasing age-dependent gene expression in photoreceptors (Hall et al., 2017; Jauregui-Lozano et al., 2022a), suggesting that transcription elongation might become less effective with advanced age. If so, we would expect that knockdown of those factors involved in transcription elongation in photoreceptors would mimic the gene expression changes observed in aging photoreceptors. To test this, we examined gene expression in photoreceptors from D30 flies expressing RNAi against nine of the identified factors involved in different stages of transcription including elongation: *Cnc*, *Cdk12*, *dom*, *Spt5*, *Spt6*, *Taf1*, *TH1*, *Top1*, *Trl* (RNAi lines used for RNA-seq highlighted in red in Figures 3A, B). As a control, we expressed RNAi against *LexA,* which did not exhibit any retinal degeneration by D30. We selected RNAi lines for *dom*, *Spt5*, and *Cdk12* that had been validated in previous studies (Li et al., 2016; Qiu and Gilmour, 2017; Scacchetti et al., 2020), and showed that the other RNAi lines could induce a significant knockdown of their target gene using qPCR (Supplementary Table S2).

We used our previously described photoreceptor nuclei-immunoenrichment (NIE) approach (Jauregui-Lozano et al., 2021) to isolate nuclear RNA from photoreceptors in *Rh1-GFP* ^*KASH*^ *, Rh1-Gal4>shRNA* flies (n = 3; Figure 4A). We note that all RNAi lines selected for RNA-seq were *shRNA* VALIUM20 lines, so we did not express Dcr in these flies. The NIE protocol isolates nuclei that are tagged with GFP^KASH^, therefore enriching nuclei from photoreceptors that have not yet degenerated, enabling us to identify the changes in gene expression in these cells. However, we also examined a small number of the flies used for each RNA-seq experiment by optical neutralization and did not observe significant retinal degeneration at D30 in this background, suggesting that the flies used for the RNAi screen were a more sensitized genetic background.

**FIGURE 4:**
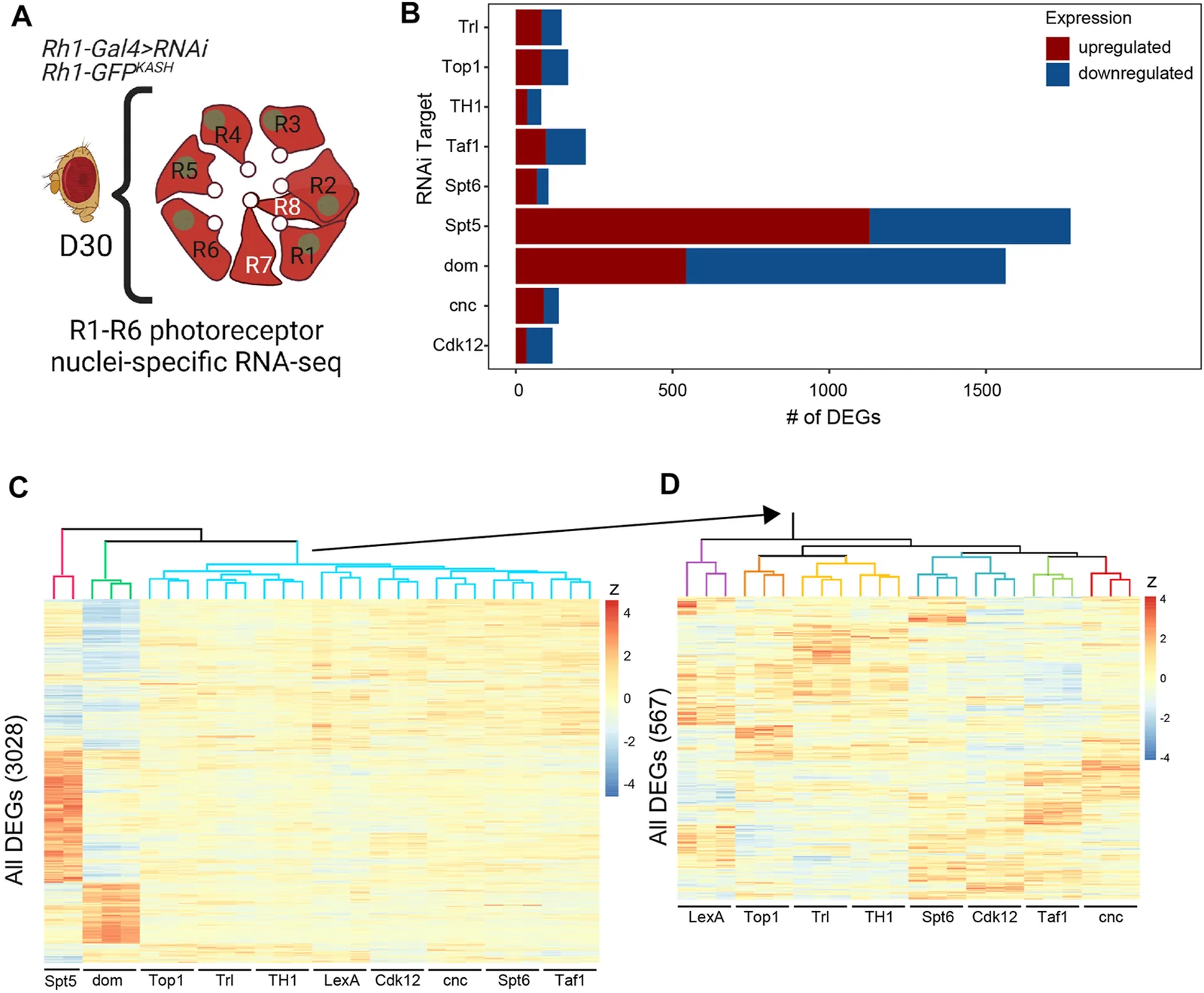
Knockdown of the factors required for photoreceptor survival leads to distinct and overlapping changes in gene expression in photoreceptors. **(A)** Photoreceptor nuclear RNA-seq was performed in D30 flies expressing *shRNA* against nine of the unique targets identified as being necessary for photoreceptor survival. **(B)** Bar plot showing the number of significantly differentially expressed genes (DEGs, FDR <0.05) identified for each *shRNA* target relative to the *shLexA* control. Up- and down-regulated genes are indicated in red and blue, respectively. **(C)** Heatmap depicting the relative gene expression (z-score) across all samples for all 3,028 DEGs identified in any *shRNA* line versus control, clustered by genes (rows) and samples (columns). The sample dendrogram is colored to show major groups. **(D)** Heatmap showing the relative expression of the 567 DEGs identified in the blue cluster in panel C.

Next, we identified differentially expressed genes (DEGs, FDR <0.05) in each RNAi line relative to the LexA control. Although we only identified a relatively small number of DEGs (<250) in many of the RNAi lines (*Trl, Top1, TH1, Taf1, Spt6, cnc, Cdk12*), knockdown of *dom* and *Spt5* had much more widespread effects on gene expression with 1,563 and 1,129 DEGs, respectively (Supplementary Table S3) Figures 4B. When we clustered the samples based on their relative expression (z score) for all DEGs, we found that both *Spt5* (red cluster) and *dom* (green cluster) samples were distinct from one another, and grouped separately from all other samples (Figure 4C). When we repeated this clustering without *Spt5* and *dom* to examine the relationship between the other samples more closely, we found that several factors showed similar changes in gene expression such as *Tr1* and *TH1* (yellow), or *Spt6* and *Cdk12* (blue), suggesting that these factors might regulate common sets of genes Figure 4D.

We next asked how the gene expression changes resulting from knockdown of these factors compared with those observed during normal aging in photoreceptors. To do this, we first performed functional enrichment analysis on the DEGs identified upon knockdown of the nine lines tested at D30 and compared this with age-dependent DEG sets previously identified in D50 and D60 photoreceptors relative to D10 (Jauregui-Lozano et al., 2022b). We separated DEGs into up- or down-regulated genes, and then identified enriched Gene Ontology terms (GO, FDR <0.05) for each DEG set and compared these between gene sets using dot plots (Figures 5A, B). We did not identify any enriched GO terms in the DEGs for *Trl*, *TH1,* or *cnc*, likely due to the relatively low number of DEGs in these samples. As suggested by the gene expression heatmaps, we observed overlapping and distinct GO terms enriched for *Spt5* and *dom* both for up- and down-regulated genes (Figures 5A, B). In addition, we observed a small number of overlapping GO terms between *Cdk12*, *Spt6*, and *Taf1* only in the downregulated genes (Figure 5B). When we compared each of the RNAi lines with the age-dependent changes in gene expression at D50 or D60, we observed that many of these age-dependent GO terms were shared with either *Spt5* or *dom*. *Spt5* and *dom* knockdown mimicked the aging DEGs for functional categories such as synapse activity, negative regulation of signaling, and growth. Knockdown of *dom* resembled aging even more extensively with commonly enriched GO terms including nervous system development, rhythmic processes, and responses to abiotic stress and regulation of the immune system. These data suggested that decreased levels of *Spt5* and *dom* resulted in similar gene expression changes at D30 to those observed in much older flies at D50 or D60. To test this, we directly compared the up- and downregulated DEGs identified upon *Spt5* or *dom* knockdown with the aging DEGs identified at D60. We observed a significant overlap of up- (Figure 6A) and downregulated (Figure 6B) genes at D60 with both *Spt5* and *dom* knockdown at D30. Strikingly, around half of all differentially expressed genes in *dom* RNAi were similarly differentially expressed in D60 flies. Moreover, both *Spt5* and *dom* were more similar to D60 than to each other. Despite this, we did observe 66 up- and 122 downregulated genes that were common between all three data sets.

**FIGURE 5:**
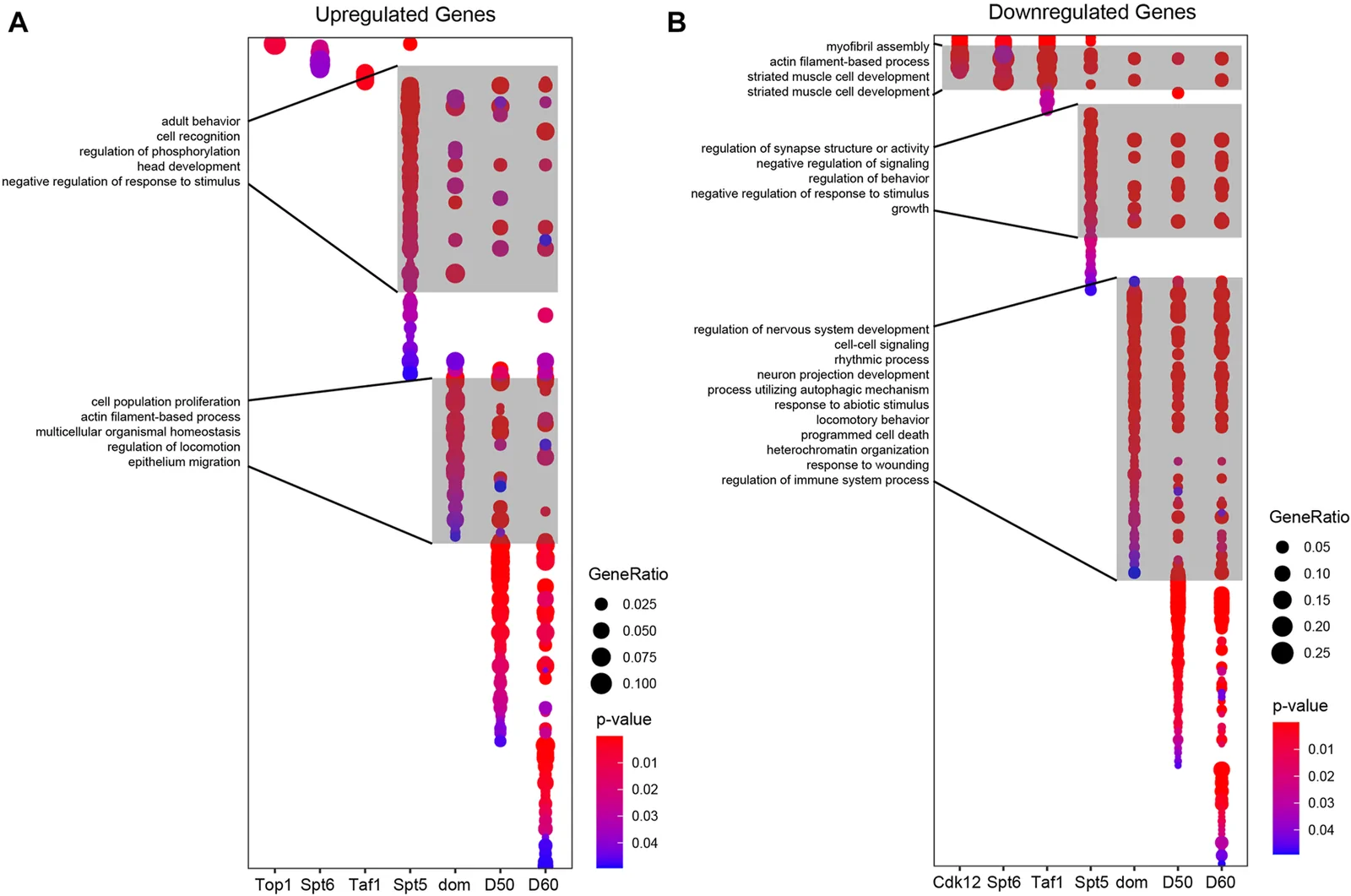
Overlap of GO terms between *Spt5* knockdown, *dom* knockdown, and aging. Dot plots depicting significantly enriched GO terms from up- **(A)** or down- **(B)** regulated genes identified in the indicated *shRNA* lines compared with age-dependent changes in gene expression in photoreceptors between D10 and D30, D50, or D60. Selected GO term labels are shown.

**FIGURE 6:**
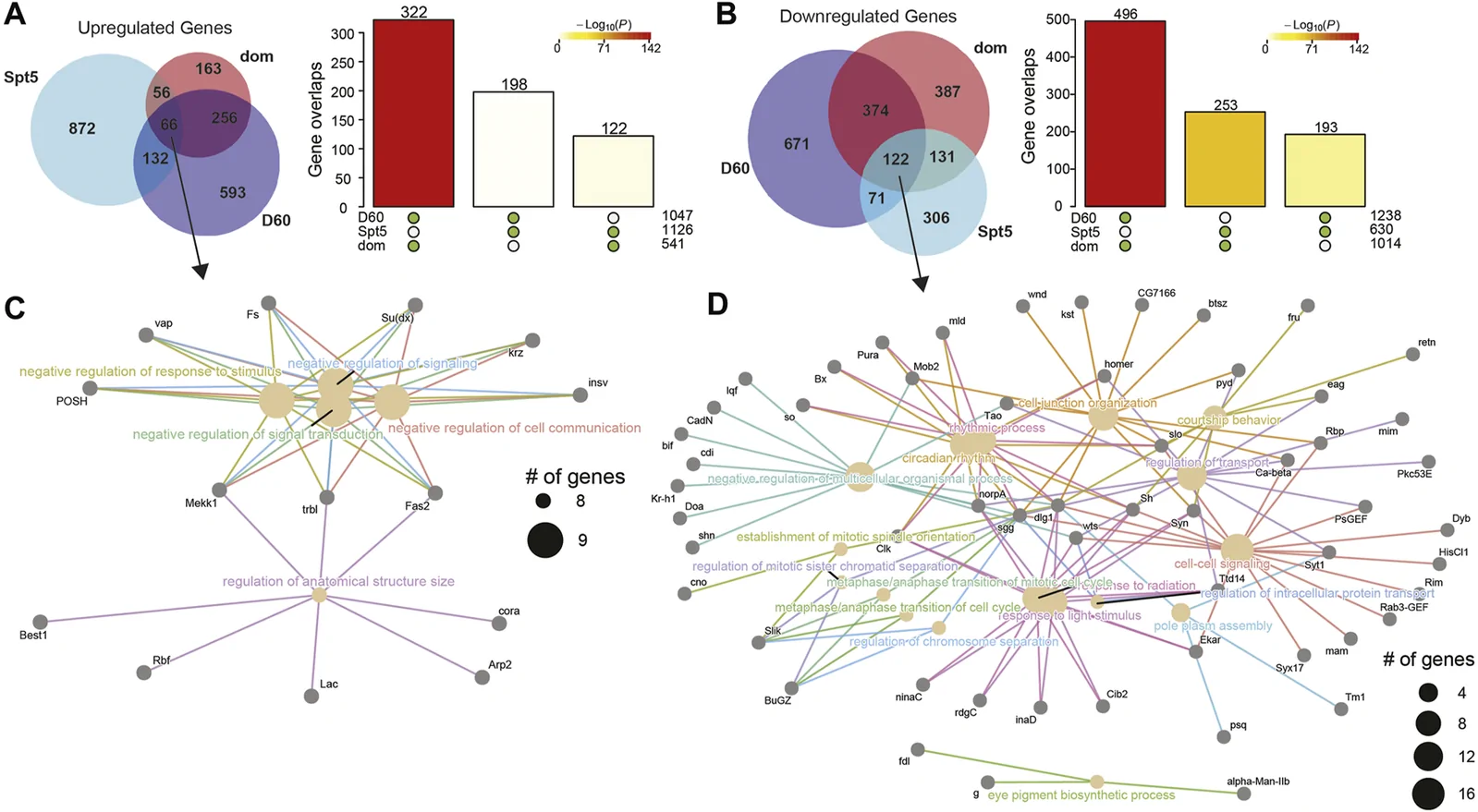
DEGs in photoreceptors with knockdown of *Spt5* and *dom* significantly overlap age-dependent DEGs in old flies. Venn diagrams of the overlap between up-**(A)** or down-**(B)** regulated gene sets upon knockdown of *Spt5* or *dom* or in old D60 photoreceptors. Bar plots show the significance of pairwise overlaps between gene sets as determined by Fisher’s exact test, colored by *p*-value. The green dots below bars indicate the gene sets for each pairwise overlap. **(C, D)** Cnet plots of up-**(C)** or downregulated **(D)** genes that overlap between *Spt5* knockdown, *dom* knockdown, and D60 photoreceptors.

To examine these common sets of genes more closely, we generated cnet plots, which display the DEGs that contribute to the enriched GO terms (Figures 6C, D). We observed a much broader group of enriched GO terms in the common downregulated genes relative to the upregulated genes, including response to light stimulus, circadian rhythm, and protein transport. Since *Spt5* and *dom*, like many of the other transcriptional regulatory factors identified in our RNAi screen, are primarily involved in transcription activation rather than repression, these common downregulated genes are likely to represent their direct targets. Many genes downregulated in *Spt5* RNAi, *dom* RNAi, and aging are involved in neuronal function, including the enzyme responsible for generating the neurotransmitter histamine, Histidine decarboxylase (*Hdc*) (Burg et al., 1993), the potassium channel component Shaker (*Sh*) (Ueda and Wu, 2006), the calcium sensor protein involved in neurotransmitter release, Synaptotagmin 1 (*Syt1*) (Littleton et al., 1993), and the calcium-dependent cadherin involved in photoreceptor axon guidance, Cadherin-N (*CadN*) (Prakash et al., 2005). Together, these data suggest the loss of *Spt5* and *dom* mimic the gene expression changes seen in older D60 flies, resulting in a premature aging transcriptional signature that includes the downregulation of neuronal-specific genes and dysregulation of circadian rhythms.

## Discussion

Here, we show that the knockdown of factors that regulate the expression of age-dependent genes in photoreceptors results in premature retinal degeneration. These data suggest that maintaining proper gene expression programs is critical for the survival of aging photoreceptors. Although transcriptional regulators that are involved in gene repression were well represented in our initial set of 155 RNAi lines, no epigenetic factors involved in repression were identified in our 18 factors that are necessary for age-dependent photoreceptor survival. We do note, however, since we used the *Rh1-ffluc* reporter as a proxy for photoreceptor number in the RNAi screen, our data may also provide information on potential transcriptional regulators of *Rh1* expression in adult photoreceptors. For example, we identified that the knockdown of transcription factors *cyc*, *CtBP,* and *Utx* resulted in increased luciferase activity relative to control, suggesting a potential role for these factors in repressing *Rh1* expression (Supplementary Table S1). These data also demonstrate that our RNAi screen was capable of detecting repressors, and support our conclusion that old photoreceptors might be most sensitive to decreased activity of the transcriptional mechanisms that promote, rather than repress, gene expression. Supporting this idea, there is a global decrease in chromatin accessibility in aging *Drosophila* photoreceptors (Jauregui-Lozano et al., 2023), and histone marks associated with active transcription show global genome-wide decreases in aging fly heads (Wood et al., 2010; Wood and Helfand, 2013). While our screen has broadly identified activators of transcription, previous studies in mouse iPSCs (Liu et al., 2011; Miller et al., 2013) and models of rapid aging disorders (Zhang et al., 2015) show that the de-repression of heterochromatin may be a driving force of aging. *Drosophila* neurons also have increased activity of transposable elements (Li et al., 2013), which have also been implicated in various human neurodegenerative diseases (Li et al., 2012). This suggests that the loss of heterochromatin with age may lead to de-repression of these elements (Andrenacci et al., 2020), and their ensuing activity may lead to increased genomic instability detrimental to aging neurons. Our data suggest that loss of heterochromatin might be less critical for photoreceptors in *Drosophila* relative to other neurons, but could also reflect that our RNAi knockdown approach would identify factors whose levels, rather than presence or absence, contribute to photoreceptor survival.

What, then, might be the critical gene expression targets that contribute to photoreceptor death? When we looked at significantly enriched GO terms in the DEG sets for all 9 of our RNA-seq lines we observed common enrichment of pathways involved in actin and myosin assembly and muscle cell development in the genes that were downregulated for *Cdk12, Spt6, Taf1, Spt5,* and *dom*. These pathways were also downregulated in old photoreceptors (Jauregui-Lozano et al., 2022b). Age-dependent changes in the cytoskeleton have been identified in several model organisms (Kounakis and Tavernarakis, 2019) and have been identified as an important modulator of neurodegenerative disease (McMurray, 2000; Cairns et al., 2004; Muñoz-Lasso et al., 2020). Moreover, when we compared the gene expression changes resulting from *Spt5* and *dom* RNAi with those observed in old photoreceptors, we found that 50% of the DEGs in *Spt5* and *dom* were also differentially expressed in old photoreceptors, with significant overlaps between all three DEG sets. Examination of the genes represented in our cnet plots (Figures 6C, D) revealed that many of the neuronal-specific processes that we previously showed are downregulated with age in photoreceptors (Jauregui-Lozano et al., 2022b) require *dom* and *Spt5*, such as response to light, and regulation of intracellular protein transport. Surprisingly, genes involved in circadian rhythm were also highly enriched in this common set of genes; we recently showed that disruption of the circadian clock in photoreceptors by expression of dominant-negative Clock transcription factor results in altered chromatin accessibility, decreased expression of phototransduction genes, and retinal degeneration (Jauregui-Lozano et al., 2022b). The unexpected finding that *dom* and *Spt5* knockdown also decreases expression of circadian genes, similar to the pattern observed in old photoreceptors, suggests a potential role for these transcription regulatory factors in this process.

At the molecular level, Spt5*,* one-half of the highly conserved DSIF complex (Wada et al., 1998), is involved in stabilizing promoter proximally paused Pol II (Andrulis et al., 2000; Moorefield, 2021). Recent findings have shown that Spt5 prevents ubiquitination and degradation of paused Pol II, and temporal knockdown in human cells results in decreased activation of target genes, and alterations in chromatin state (Aoi et al., 2021; Hu et al., 2021). In addition to stabilizing paused Pol II, Spt5 regulates the release of the paused Pol II through its interaction with NELF (Hu et al., 2021). Further, Spt5 has additional roles later in transcription elongation because its loss results in inefficient transcription termination where transcription proceeds beyond the normal transcription end site (Baejen et al., 2017; Fitz et al., 2018). Beyond its roles in regulating Pol II pausing, Spt5 contributes to the recruitment of the Paf1 complex, a transcription elongation factor which in turn recruits several other chromatin-modifying complexes including the H3K4me3 methylase COMPASS, Rad6-Bre1, and Set2/SETD2 (Adelman et al., 2006; Mayekar et al., 2013; Wier et al., 2013; Song and Chen, 2022). Thus, loss of Spt5 function in *Saccharomyces cerevisiae* is accompanied by bulk decreases in levels of the active H3K4me3 and H3K36me3 marks deposited by Set1/COMPASS and Set2, respectively (Zhou et al., 2009). We observed decreased genome-wide levels of H3K4me3 and H3K36me3 in aging photoreceptors (Jauregui-Lozano et al., 2023), potentially suggesting that Spt5-dependent transcription activation becomes less efficient with age. However, it is unclear whether aging affects Spt5 activity itself, or an upstream or downstream component of this interconnected transcriptional mechanism. Thus, while our data indicate that genes regulated by Spt5 are susceptible to age-associated transcriptional changes, it remains to be determined mechanistically how aging disrupts this regulation.

The *dom* gene encodes the only SWR1-like ATP-dependent chromatin remodeler ortholog in flies (Börner and Becker, 2016). Knockdown of *dom* would disrupt its function both in the NuA4/Tip60 or SWR1-like complex (Squatrito et al., 2006; Scacchetti et al., 2020), and the RNAi line used to target *dom* for RNA-seq analysis targets the isoform that is associated with the SWR-1 like complex, hinting that this might be the relevant target. However, the activity of the dom isoform associated with the NuA4/Tip60 complex has been shown to regulate circadian genes in specialized neurons in the brain (Liu et al., 2019), and we also identified the Tip60 HAT within the NuA4/Tip60 complex in our RNAi screen, suggesting that both the NuA4/Tip60 and SWR1-like complexes could be involved in these age-dependent gene expression changes. In *Drosophila*, the histone variant H2A.V takes on the roles of both histone variants H2A.X and H2A.Z (Baldi and Becker, 2013; Liu et al., 2019). The *domino* isoform associated with the SWR-1 like complex is speculated to be responsible for bulk H2A.V incorporation into chromatin, while the isoform associated with NuA4/Tip60 targets H2A.V incorporation to specific loci (Liu et al., 2019). During aging, DNA damage accumulates, resulting in Pol II stalling during elongation and an overall reduction of transcription (Gyenis et al., 2023). Since H2A.V serves as a mark for DNA damage (Baldi and Becker, 2013), it is possible that loss of *dom* could impair H2A.V incorporation in photoreceptors and disrupt DNA damage repair. Thus, *dom* knockdown could lead to age-associated transcriptional changes because it mimics DNA damage-associated Pol II stalling, although this mechanism has not been tested in this study.

Although the changes in gene expression observed in the *Spt5* and *dom* knockdowns resemble those in old photoreceptors, the mechanism underlying a potential decrease in the activity of these transcriptional regulators remains unclear. Neither *Spt5*, *dom*, *Tip60*, nor any of the other 18 genes identified in our screen are downregulated during aging at the nuclear transcript level when comparing D50 and D60 flies to D10 flies (Jauregui-Lozano et al., 2022b). Also, none of these genes with detectable protein levels in *Drosophila* heads or eyes have age-dependent changes in abundance (Hall et al., 2021). Thus, although *Spt5* and *dom* knockdown resemble the transcriptional signature of aging photoreceptors, this is unlikely to be simply due to decreased expression of these proteins. Although it is possible that the activity of Spt5 and dom themselves decrease, potentially due to regulation of other components of the multi-subunit complexes or post-translational modification, it is also possible that their overlap with aging reflects alterations in activity of the overall pathways in which they function. In this second model, knockdown of Spt5 or dom results in premature photoreceptor degeneration because the pathway in which they function has become defective with age. Overall, these data provide a starting point for identifying the mechanisms that lead to altered gene expression in aging photoreceptors, and potentially other neuronal cells, and demonstrate that maintaining active gene expression mechanisms is critical for the survival of these long-lived cells during aging.

## Materials and methods

### Fly strains, genetics, and aging

Flies were raised in 12 h:12 h light:dark conditions at 25°C on standard fly food as previously described (Jauregui-Lozano et al., 2022a). Aging was conducted by collecting flies 3 days after eclosion so that flies were ± 1 day old. Flies were transferred onto fresh food every 2–3 days. Male flies were used for all experiments. All genotypes used in this study are described in Supplementary Table S4. To assess knockdown efficiency, RNAi lines were crossed to *Act5C-Gal4,* and third instar larvae were collected for analysis. If ubiquitous knockdown led to developmental lethality or if RNAi stocks had balancer chromosomes, RNAi lines were crossed to *Act5C-Gal4, tub-Gal80* ^*ts*^ flies, progeny raised at 18°C and shifted to 29°C for 24 h at D5 post-eclosion, and adult males collected for analysis. The flies used for RNA-seq analysis were characterized previously in our lab and contain the *Rh1-GFP* ^*KASH*^ transgene inserted in the 5′ UTR of the *Catalase* gene, which increases nuclear *Catalase* RNA levels but does not substantially increase steady-state *Catalase* mRNA levels (Escobedo et al., 2022). In addition, when we checked the steady-state mRNA level of these flies in the background used for this study (red-eyed background) by qRT-PCR, we found no significant change in *Catalase* mRNA levels relative to a control fly line with the *Rh1-GFP* ^*KASH*^ transgene inserted at a different locus (data not shown).

### Luciferase assays and optic neutralization

Luciferase assays and optic neutralization were performed as previously described (Stegeman et al., 2018). For optic neutralization, we blindly assessed retinal degeneration scores using a scale from 1 to 7 based on all in-focus ommatidia.

### Statistics

All statistical tests were performed using R or Microsoft Excel. Significant changes in luciferase activity or optic scores were compared between RNAi lines and all four control lines using Dunnett’s test. The significance threshold for the initial RNAi lines (shRNA #1) was set at a *p*-value <0.05 for both luciferase and optic, and at *p* < 0.05 and *p* < 0.01 for the second independent RNAi line (shRNA #2) for luciferase activity and optic scores, respectively. Statistical analysis for aging time course data (luciferase activity and optic neutralization) was conducted using ANOVA with post hoc Tukey HSD. All other pairwise statistical analyses were conducted by Students t-test with a significance threshold of *p*-value <0.05.

### Photoreceptor-specific nuclei-immunoenrichment (NIE)

NIE was conducted as previously described (Jauregui-Lozano, et al., 2021) using 150—250 male D30 flies. A full step-by-step protocol is available at: dx.doi.org/10.17504/protocols.io.buiqnudw.

### RNA-seq and bioinformatics

RNA-seq libraries were constructed and differential gene expression analysis was conducted using EdgeR as previously described (Escobedo, et al., 2022) with the following minor modifications. Counts were filtered to remove lowly expressed genes requiring counts per million (cpm) > 30 in at least 27 of the 29 libraries (10 RNAi lines, n = 3) resulting in 7,069 genes used for downstream analysis. We discarded one replicate for *Spt5* due to poor quality. RUV-seq normalization was conducted using a k = 4 to account for the variation between the 10 RNAi lines, as well as any variation due to batch effects. All plots were generated in RStudio (v. 4.0.5) using custom scripts. Gene Ontology (GO) enrichment was conducted using Cluster profiler (v3.18.1) (Yu et al., 2012).

## Data Availability

RNA-seq data for the RNAi screen lines is available at the Gene Expression Omnibus (GEO) repository using GSE225499. The aging RNA-seq data used for comparisons is available at GSE169328 and GSE174515.
